# Determinants of Genomic RNA Encapsidation in the *Saccharomyces cerevisiae* Long Terminal Repeat Retrotransposons Ty1 and Ty3

**DOI:** 10.3390/v8070193

**Published:** 2016-07-14

**Authors:** Katarzyna Pachulska-Wieczorek, Stuart F.J. Le Grice, Katarzyna J. Purzycka

**Affiliations:** 1 Laboratory of Structural Chemistry and Biology of Nucleic Acids, Institute of Bioorganic Chemistry, Polish Academy of Sciences, Poznan 61-704, Poland; kasiapw@ibch.poznan.pl; 2 RT Biochemistry Section, Basic Research Laboratory, National Cancer Institute, Frederick, MD 21702, USA

**Keywords:** Ty1 retrotransposon, Ty3 retrotransposon, RNA packaging, RNA trafficking, Gag protein

## Abstract

Long-terminal repeat (LTR) retrotransposons are transposable genetic elements that replicate intracellularly, and can be considered progenitors of retroviruses. Ty1 and Ty3 are the most extensively characterized LTR retrotransposons whose RNA genomes provide the template for both protein translation and genomic RNA that is packaged into virus-like particles (VLPs) and reverse transcribed. Genomic RNAs are not divided into separate pools of translated and packaged RNAs, therefore their trafficking and packaging into VLPs requires an equilibrium between competing events. In this review, we focus on Ty1 and Ty3 genomic RNA trafficking and packaging as essential steps of retrotransposon propagation. We summarize the existing knowledge on genomic RNA sequences and structures essential to these processes, the role of Gag proteins in repression of genomic RNA translation, delivery to VLP assembly sites, and encapsidation.

## 1. Introduction

Long-terminal repeat (LTR) retrotransposons are transposable genetic elements that comprise a significant fraction of many eukaryotic genomes [1,2]. They are progenitors of retroviruses, differing in that they replicate intracellularly [3,4,5]. Like retroviruses, LTR-retrotransposons replicate via an RNA intermediate and insert their double-stranded DNA into the host genome [6,7,8]. Retrotransposons play beneficial roles in host genome remodeling and evolution, but also cause mutations and insertional gene inactivation that can lead to diverse genetic diseases [9,10]. Our knowledge of LTR-retransposons is mostly based on studies of Ty elements of *Saccharomyces cerevisiae.* Ty1 and Ty3 are the most extensively characterized LTR-retrotransposons (see recent reviews [11,12]). Ty1 represents the *Pseudoviridae* family and is the most abundant mobile genetic element in the genome of *S. cerevisiae* [13,14]. Ty3 belongs to *Metaviridae* family of LTR-retrotransposons, whose members are more related to retroviruses than *Pseudoviridae* in genome organization and sequences of their encoded proteins [1,3,15]. In haploid yeast cells, Ty3 RNA is present at low levels and its expression is induced by pheromone stimulation in mating yeast cells [16,17,18]. Full-length Ty1 or Ty3 genomic RNA (gRNA) plays dual role in replication and serves as a template for translation [19] as well as the retrotransposon genome that is packaged into virus like particles (VLP) and reverse transcribed [6,8]. Therefore a fine balance between gRNA translation and packaging events is required for productive retrotransposition.

## 2. Genetic Organization and Replication Cycle of Ty Retrotransposons

**Figure 1:**
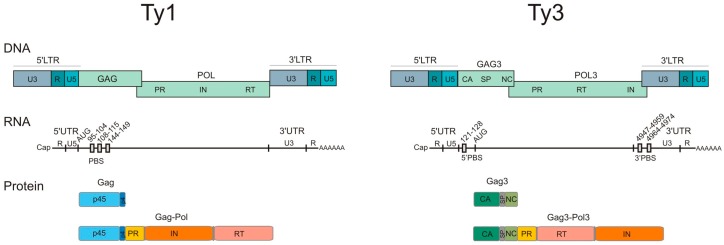
Structure of the Ty1 and Ty3 elements, RNAs, and proteins. Both elements contain long terminal repeats (LTRs) and central coding region with two overlapping ORFs: *GAG* and *POL*. The capped and polyadenylated genomic RNA contains untranslated regions (UTRs) and a primer binding site (PBS) that binds initiator tRNAiMet. *GAG* and *POL* encode Gag and Gag-Pol. Gag-Pol polyprotein contains enzymatic proteins: protease (PR), reverse transcriptase (RT), and integrase (IN). Ty1 Gag is post-translationally cleaved into Gag p45 and short peptide p4, while Ty3 Gag3 is cleaved to capsid (CA), short spacer (SP), and nucleocapsid (NC).

Ty1 and Ty3 share many important structural and functional characteristics. The elements are similar in size; integrated full-length Ty1 is 5.9 kb, and the Ty3 element is 5.4 kb in length. Both elements contain two overlapping open reading frames (*ORFs*), *GAG* and *POL*, flanked by long terminal repeats (LTRs) [7,17,20,21] (Figure 1). The *GAG* *ORF* encodes the Gag capsid-like protein, while the *POL* *ORF* encodes enzymes required for Gag and Gag-Pol maturation (protease (PR)), retrotransposon replication (reverse transcriptase (RT)) and integration into the host genome (integrase (IN)). Organization of the Ty3 *POL3 ORF* is akin to that of retroviruses (PR-RT-IN) while the Ty1 *POL ORF s*equence order is PR-IN-RT [22,23,24].

Retrotransposons exploit cellular machinery to transcribe their genomic copy, and both Ty1 and Ty3 transcripts synthesized by RNA polymerase II are capped and polyadenylated before nuclear export. Despite the fact that Ty transposition is a rare event, Ty1 RNA comprises up to ~10% of polyadenylated mRNA in haploid *S. cerevisiae* cells [6,25,26]. This probably results from significantly longer half-life of Ty1 RNA compared to yeast mRNAs [27] and recent data indicate that Ty1 Gag is required for gRNA stability [28]. Ty1 and Ty3 genomic transcripts (5.7 kb and 5.2 kb, respectively) are similarly organized and each contains two partially overlapping *GAG* and *POL* open reading frames flanked by untranslated regions (UTR) at the 5′ and 3′ termini [7,17,20,24] (Figure 1). The 5′ UTR is comprised of unique U5 sequence and R region, the latter of which is repeated in the 3′ UTR. In addition to R, the 3′ UTR contains a unique U3 sequence. Splicing of Ty1 and Ty3 transcripts has not been detected, but the presence of shorter transcripts is documented [29,30,31,32]. After export to the cytoplasm, full-length RNAs provide the template for both protein translation and gRNA that is ultimately packaged into VLPs. The primary translation products are Gag and Gag-Pol precursors, the latter resulting from a +1 ribosomal frameshifting event. Gag-Pol is produced at the 5% level of that of Gag [33,34,35,36]. Ty1 and Ty3 frameshifting results from translational pausing due to the presence of rare tRNA codons [35,37,38,39].

**Figure 2:**
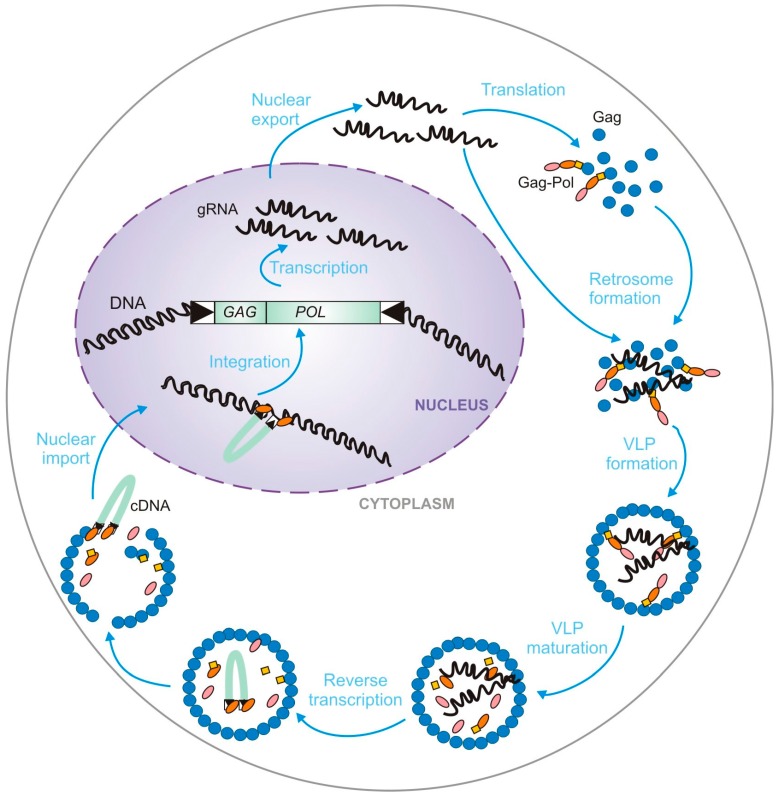
Ty replication cycle with the major steps of retrotransposition outlined. Ty element is transcribed by host cell machinery. The RNA is exported to the cytoplasm and translated into Gag and Gag-Pol. Ty RNA, Gag, and Gag-Pol associate to form retrosomes where dimeric Ty genomic RNA and tRNAiMet are encapsidated into the VLP. Cleavage of Gag and Gag-Pol proteins by the Ty protease leads to VLP maturation. Ty RNA is reverse transcribed and cDNA is integrated into the host genome.

The Ty1 and Ty3 gRNA, Gag, and Gag-Pol precursors colocalize in specific cytoplasmic foci termed retrosomes, where they assemble into VLPs [40,41,42,43] (Figure 2). VLPs contain a Gag structural protein, the *POL*-encoded enzymes, and two copies of Ty gRNA organized in a dimeric form [44,45]. Once VLPs undergo maturation due to the specific Gag and Gag-Pol proteolysis, dimeric gRNA is reverse transcribed into (−) DNA, utilizing cellular tRNAiMet as a primer [46,47,48]. tRNAiMet is packaged into VLPs during assembly. Both elements contain a bipartite primer binding site (PBS) but Ty1 PBS is localized in *GAG* ORF while the 5′ and 3′ portions of the Ty3 PBS are located at opposite ends of the genome [47,48] (Figure 1). After (+) strand DNA synthesis nuclear import occurs and a nuclear localization signal (NLS) is present on IN [49,50]. The replication cycle is completed by integration into regions of the genome that are associated with transcription by RNA polymerase III [51,52,53,54] (Figure 2). Several host factors required for Pol III activity are important determinants of target sequence specificity [54,55,56,57,58] and recent data indicate that an interaction between Ty1 IN and the AC40 subunit of Pol III plays the predominant role in targeting Ty1 integration upstream of genes transcribed by RNA Pol III [59].

## 3. *Cis*-Acting Sequences in Retrotransposons gRNA

Retroelement gRNAs contain internal structures fundamental to propagation. Prominent among these motifs are *cis*-acting sequences required for gRNA dimerization, packaging, and priming of reverse transcription. The *cis*-acting sequences were first delimited for Ty1 using a mini-Ty1 donor-helper system [60]. Mini-Ty1s are deletion mutants of the Ty1-H3 element (a His+ revertant; [6]) lacking functional gene products. Helper elements are incapable of retrotransposition but supplement Ty1 proteins in *trans*, enabling mini-Ty1s containing *cis* sequences to transpose. These studies demonstrated that regions located within or adjacent to the LTRs are important while up to 5 kb of Ty1 internal sequences can be deleted without any significant effect on transposition. The minimal Ty1 element capable of retrotransposition when proteins are supplemented in *trans* contains 380 nt of 5′-end of the (+) RNA genome and 357 nt from its 3′-end. Using a similar assay, *cis*-acting sequences in Ty3 RNA were delimited at the 5′ and 3′-ends, and nt 429 to 4979 were demonstrated dispensable for retrotransposition when proteins were supplemented in *trans* [12,36].

**Figure 3:**
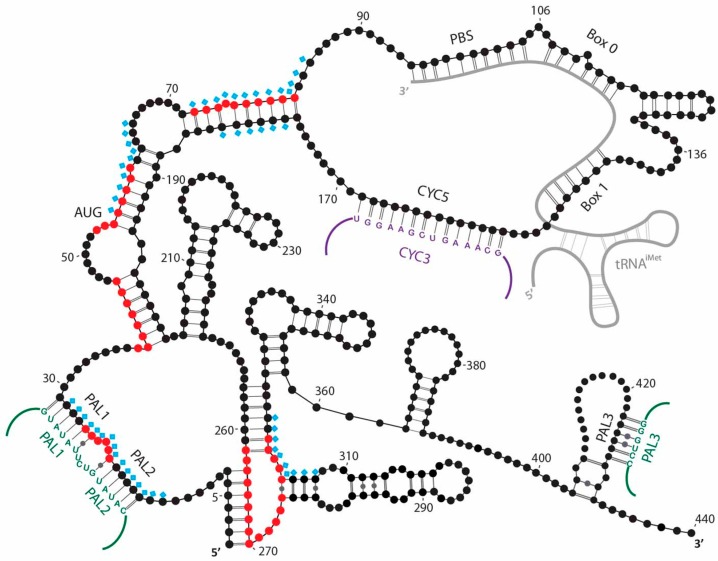
Secondary structure model of the 5′ end of Ty1 genomic RNA derived from structural analysis in different biological states by selective 2'-hydroxyl acylation analyzed by primer extension (SHAPE) [61]. Nucleotide positions at which SHAPE reactivities increased when proteins were gently removed from VLP-associated Ty1 genomic RNA are marked with blue diamonds. Those positions most likely correspond to Gag binding sites within the dimeric Ty1 genomic RNA in VLPs. Regions in monomeric Ty1 RNA mapped in vitro as binding sites for Gag C-terminal region are represented in red [62]. Palindromic (PAL) sequences, including reciprocal interstrand interactions are annotated in green [61], and cyclization mediated by CYC5 and CYC3 in violet [63].

tRNAiMet is essential for Ty1 and Ty3 retrotransposition. Using mutational analyses Ty1 gRNA sequences required for primer binding were located between nt 94 and 104 [46] based on complementarity to the tRNAiMet 3′ acceptor stem. However, this 10nt Ty1 PBS sequence was not required for tRNAiMet packaging into VLPs [46]. Further studies presented additional evidence for sequences located 3′ to PBS that were necessary for tRNAiMet encapsidation [64,65]. Those regions of complementarity to the tRNAiMet TΨC and DHU arms not only enable primer packaging, but also play a role in the initiation of reverse transcription in vivo [64,66]. Similar molecular determinants for retrotransposition were found in the tRNAiMet primer for Ty1 and Ty3 [48]. Although both elements contain a bipartite PBS formed from three segments, they are differently organized. Ty1 PBS is localized in *GAG* ORF and the largest region separating its PBS sequences is 28-nt, while parts of the Ty3 PBS are located at the opposite ends of the genome, adjacent to the 5′ UTR (one segment, nt 121–128) and in the 3’ UTR (two segments) [47,48]. In Ty3, tRNAiMet annealed to opposite ends of gRNA is proposed to mediate its cyclization [47]. For both Ty1 and Ty3, an interaction between gRNA ends is required for efficient initiation of reverse transcription [47,63]. Ty1 cyclization is not mediated by the tRNAiMet bridge, but occurs via direct interaction of complementary sequences within gRNA, namely CYC5 at the 5′-end and CYC3 at the 3′-end [63] (Figure 3). The CYC5 sequence is located downstream of the PBS and, interestingly, is also complementary to the tRNAiMet DHU stem, raising the possibility for additional interactions [64]. However, defects in initiation of reverse transcription caused by mutations compromising CYC pairing could be complemented by mutations that restored CYC5:CYC3 pairing, proving their direct interaction [63]. In studies using the modular mini-Ty1 system (pJEF1254; [60]), Bolton et al. demonstrated that another long-range intramolecular interaction was critical for Ty1 propagation [67]. In particular, base-pairing between the 1-GAGGAGA-7 sequence within the 5′ repeat (R) region and the 264-UCUCCUC-270 sequence downstream of the PBS was required for efficient initiation of reverse transcription. We further defined this region as constituting a part of an RNA intramolecular pseudoknot by combining chemical probing with mutational analysis [68] (Figure 3). The structure of Ty3 RNA remains uncharacterized while a comprehensive model of Ty1 secondary structure in VLPs and in vitro was determined [61]. The 5′-end of Ty1 gRNA is highly structured and compactly folded, analogous to retroviral 5′ UTRs [69,70]. Interestingly, UTRs of yeast RNAs are less structured than corresponding coding regions [71].

## 4. Retrotransposon Gag Proteins

Gag and its mature products are the major VLP structural components, serving as multifunctional regulators that orchestrate retrotransposon replication. Organization of the 290-amino acid Ty3 Gag3 is related to the counterpart proteins from simple retroviruses encoding a capsid (CA) and nucleocapsid (NC) domain, separated by short spacer (SP) (Figure 1). CA is critical for Ty3 Gag3 multimerization, whereas NC is required for all nucleoprotein interactions mediated by Ty3 Gag3 [36,72,73,74]. Mature Ty3 NC resembles retroviral NC proteins and is small (57-aa), basic (pI = 11.15) protein, containing one CCHC zinc-finger (ZF) motif [43,75,76]. Some members of *Orthoretrovirinae* subfamily also have nucleocapsid protein with only one ZF (Gamma- and Epsilonretroviruses), while others possess two motifs. *Spumaretrovirinae* NC-like proteins lack ZF motifs [77]. Ty3 NC displays nucleic acid chaperone activity and promotes nucleic acid aggregation, tRNAiMet annealing, and Ty3 RNA dimerization in vitro [47,78]. During retrovirus replication Gag or NC, via chaperone activity, facilitate genome dimerization and packaging, annealing of the tRNA primer and the strand-transfer events associated with reverse transcription [79]. Based on those observations, the role of Gag3 and NC in Ty3 replication can be considered analogous to retroviral Gag and NC proteins [79,80,81,82]. Mutational studies of the Ty3 NC domain indicate that both the N-terminal basic region (NTD) and zinc-finger play important roles in association of Ty3 Gag3 with gRNA [72,78]. The NC domain controls Ty3 Gag3 and gRNA co-localization and trafficking prior to assembly, and is required for gRNA packaging into VLP. Mature NC chaperones nucleic acid interactions during reverse transcription and mutations that abolish Ty3 Gag3 processing into mature NC block cDNA synthesis [75].

Although functionally related, Ty1 Gag lacks sequence and structural homology to Ty3 or retroviral Gag proteins [83]. The 441 aa Ty1 Gag precursor undergoes only one C-terminal cleavage by PR, providing the mature 401 aa Gag and a 40 aa, acidic peptide [84,85,86,87] (Figure 1). Ty1 Gag possesses the ability to interact with RNA in vitro [19,88] but does not have the canonical NC domain with zinc-finger motif. Based on in vitro studies, the RNA binding and chaperone activity region of Ty1 Gag has been mapped to C-terminal residues Asn299–His401, a region containing three clusters of basic amino acids [89]. The importance of this C-terminal region was further demonstrated using a Ty1 mutant from which this had been deleted. This mutant fails to interact with RNA in vitro. In case of retroviral NC proteins, basic residues were likewise demonstrated important for chaperone activity, as deleting the N-terminal basic domain significantly reduced these properties [90,91,92,93,94]. A synthetic peptide (TYA1-D) corresponding to the C-terminal 103-aa region of Ty1 Gag binds RNA, promotes annealing of tRNAiMet, Ty1 RNA dimerization and initiation of reverse transcription in vitro [89]. Recently, chaperone activity has been further characterized using recombinant proteins corresponding to diverse regions of Ty1 Gag. The C-terminal region (CTR) protein encompassing the C-terminal 228 residues of Ty1 Gag, and containing three basic clusters, displayed robust chaperone activity comparable to TYA1 peptide [62], while a truncated form of CTR (sCTR) lacking 47 C-terminal residues and containing only the first and second basic cluster, lost activity. This observation supports the critical function of the third basic cluster for chaperone activity of Ty1 Gag. Alternatively, all three basic clusters are required to promote Ty1 Gag/RNA interactions.

## 5. Trafficking of Ty gRNA and Gag to Retrosomes

Ty1 and Ty3 gRNAs are not divided into separate pools of translated and packaged RNA, thus gRNA trafficking and packaging into VLPs require an equilibrium between competing events of translation and packaging. VLP assembly starts at the specific, microscopically distinct, cytoplasmic foci known as retrosomes, where gRNA and Gag colocalize [40,41,42]. Both gRNA and Gag are required for retrosome nucleation, as when Gag is not translated or gRNA is absent from the cytoplasm, retrosomes do not form [28,42]. Retrosomes are still observed in Ty1 strains with mutations in, or deletion of, PR, IN, and RT coding sequences, indicating that retrosome formation is independent of enzyme activity [28]. A direct gRNA interaction with Gag is required for retrosome nucleation and mutations in C-terminal RNA binding domain of Ty1 Gag [28,42] or NC domain of Ty3 Gag3 [72] disrupt retrosome formation.

Little is known about Ty gRNA trafficking to retrosomes and it remains unclear where the first interaction with Gag occurs. Recent findings indicate that Ty1 gRNA is translated in association with signal recognition particle (SRP), which interacts with nascent Gag polypeptide for transport into the ER [95]. The SRP pathway is universally conserved and utilized for co-translational targeting of mRNA coding secretory and membrane proteins to the ER [96]. As Ty1 gRNA is translated, SRP interacts with the ribosome and specific hydrophobic sequences in the nascent Gag polypeptide to target the translating complex to the ER, whereupon Gag enters the ER lumen and assumes a stable conformation. When the SRP is rendered genetically defective by an *srp68-DAmP* mutation, Gag is present but rapidly turned over and retrosomes do not form. Stable Ty1 Gag is retro-translocated from the ER lumen to the cytoplasm and binds translating Ty1 gRNA. Multimerization of Gag bound to Ty1 gRNA may repress gRNA translation and induce a shift from its translation to packaging into VLPs [95].

Current data raises an interesting possibility that Ty1 and Ty3 Gag proteins play a role in nuclear export of gRNAs [72,97]. Using a two-plasmid system, where Ty1 Gag and gRNA are expressed independently, Checkley et al. demonstrated that Gag enhances gRNA export from nucleus, stability, and co-localization into retrosomes [28]. In the absence of Gag, Ty1 gRNA accumulates in the nucleus and becomes unstable when the Ty1 element contains a chain terminating mutation (Ty1fs) adjacent to the Gag initiation codon. When Gag is expressed independently, Ty1fs RNA regains its stability and is present in the retrosomes. The nuclear localization signal (NLS) has not been identified in Ty1 Gag and Ty1 Gag was not shown to enter the nucleus. However, Ty1 Gag nuclear localization may be only transient and difficult to detect. Nevertheless, Ty1 Gag is proposed to accumulate at the nuclear periphery and enhance gRNA nuclear export using Mex67p pathway [28,41]. This scenario does not exclude the possibility that during retrotransposition, Ty1 gRNA may be exported from the nucleus independently of Gag via Mex67p pathway. Translation of Ty1 gRNA results in accumulation of Gag and Gag-Pol in the cytoplasm and Gag may capture newly exported Ty1 transcripts at the nuclear periphery. Unlike Ty1 Gag, Ty3 Gag3 is suggested to enter the nucleus to recruit newly transcribed gRNA for packaging. Although Ty3 wild type Gag3 is not found in the nucleus, mutating conserved residues within its NC domain that disrupt RNA binding results in Gag3 accumulation in the nucleus, a decrease of Ty3 gRNA concentration in retrosomes and reduced packaging [72]. The effects of these mutations indicate that the Gag NC domain is required for both packaging and delivery of gRNA to VLP assembly sites. Consequently, early Gag binding to gRNA, both in the nucleus or immediately after nuclear export, may sequester gRNA from translation machinery and facilitate its trafficking to the retrosomes for VLP formation. Retroviral Gag proteins, including those of HIV-1, murine leukemia virus, human and simian foamy viruses, and Rous sarcoma virus may enter the nucleus [98,99,100,101]. Moreover, nuclear trafficking of RSV Gag is required for efficient packaging of viral gRNA into assembling virus particles [102]. However, it remains unknown whether it is a property of other retroviral Gag proteins.

## 6. P-Bodies and Retrosome Formation

Many lines of evidence indicate that both Ty1 and Ty3 require mRNA processing body (P-body) proteins for effective retrotransposition and VLP assembly. Eukaryotic P-bodies are cytoplasmic ribonucleoprotein granules wherein mRNA deadenylation-dependent and nonsense-mediated decay factors are concentrated along with their mRNA substrates and where mRNA decay processes can occur [103,104,105]. Ty3 retrosomes co-localize with P-bodies [40] and the Gag3 NC domain is required for this localization [72]. Mutational studies suggest that both the N-terminal basic tail and zinc finger are engaged in association with P-body proteins [72]. P-bodies are not only sites of mRNA degradation but also play an important role in translation repression and mRNA segregation for storage or decay [106,107]. Ty3 VLPs assembly in P-bodies might be beneficial and existing results support the hypothesis that P-body factors may serve to divide the translation and assembly functions of Ty3 gRNA [40,108,109]. Association of P-body proteins may sequester Ty3 gRNA from cellular translational machinery, thus increasing the pool that is not actively translated and can be packaged. A significant fraction of Ty1 Gag foci localize in P-bodies [110], but different conditions promote Ty1 retrosome and P-body formation [41,97,110]. Nevertheless, P-body components are important cofactors of both Ty1 and Ty3 retrotransposition and their deficiency negatively influences the appearance of retrosomes, the level of retrotransposition-competent VLPs, protein maturation, and gRNA packaging [40,97,109,110,111,112].

## 7. RNA Packaging

Although Ty gRNA is selected from a pool of excess cellular RNAs and selectively packaged into VLPs [8,113], sequences important for packaging have not been precisely defined. It was demonstrated that the R region of the 5′ UTR, the entire *POL*, and 3′ UTR are dispensable for Ty1 RNA localization to retrosomes [42] while Ty3 RNA localization to retrosomes and packaging into VLPs was dependent on the presence of UTRs or *POL* sequences [108]. For efficient VLP formation in a heterologous host, Ty1 [114] or Ty3 [73] *GAG* sequence was sufficient. Based on the mini-Ty1 donor-helper system Xu and Boeke [60] demonstrated that *cis*-acting sequences required for gRNA packaging reside within 580 nt at the RNA 5′ terminus. When additional nucleotides, up to position 380, were deleted from mini-Ty1 RNAs, packaging was reduced to 80%. Further deletion of nucleotides 237–380 reduced packaging to 15% and significantly impeded the amount of RNA co-purified with VLPs. Collectively, these observations argue that either major *cis*-acting sequences required for Ty1 RNA packaging reside within 237–380 region or its presence facilitates proper exposure of packaging element. The 237–380 fragment encompasses part of the sequence essential for folding of the Ty1 RNA pseudoknot (nt 256–270) [68] (Figure 3). RNA kissing loops that can be defined as an “intermolecular pseudoknot” mediate HIV dimerization, and therefore are determinants of retrovirus packaging (for review, see [115]). Interestingly, the Ty1 RNA pseudoknot may provide a binding site for proteins within VLPs [61]. In theory, this reflects retroviral propagation where Gag binding is fundamental for RNA encapsidation [116,117,118]. Therefore, the Ty1 RNA pseudoknot was a plausible mediator of gRNA packaging. However, using a combination of structural and functional analyses, a role for the Ty1 pseudoknot in RNA encapsidation could not be demonstrated [68].

Both genetic studies and limited in vitro data imply that Ty encapsidation relies on recognition of *cis*-acting sequences in gRNA by Gag. Moreover, it is not known if the same nucleotide sequences are recognized by Gag during nuclear export, trafficking to retrosomes and gRNA packaging [28,42,60]. The sites of Gag binding within Ty1 or Ty3 gRNA are not precisely defined. Chemoenzymatic analyses of Ty1 gRNA inside mature VLPs and after gentle deproteinization identified sites occupied by proteins, but could not define the identity of the protein bound. However, since Gag is the most abundant protein in VLPs we hypothesised that the majority of those sites were occupied by Gag (Figure 3). The most prominent changes in SHAPE-determined nucleotide reactivity after extraction of proteins were observed within ~500 nt adjacent to the gRNA 5′-end indicating that disrupting RNA-protein interactions impacts mainly the Ty1 gRNA region containing the major *cis*-acting signals for dimerization, packaging, and initiation of reverse transcription [61]. A recent study confirmed and extended those findings via in vitro hydroxyl radical footprinting of Gag CTR/Ty1 RNA complex [62] (Figure 3). A major Gag CTR binding site was detected within the pseudoknot present at the 5′-end of Ty1 RNA. Interestingly, mutations disrupting pseudoknot formation interfere with retrotransposition [68,119]. Additional Gag CTR binding sites are adjacent to the Ty1 RNA sequences that are important for tRNAiMet annealing, cyclization, and possibly dimerization [61,62].

VLP-associated Ty1 and Ty3 gRNAs are dimeric [44,45,47]. Early models suggested that dimerization was mediated by non-covalent interaction of two tRNAiMet molecules hybridized to gRNA [47,89]. tRNAiMet contains a 12-nt palindromic sequence at the 5′-end which is not paired with Ty1 gRNA when tRNAiMet is annealed. This strand of tRNAiMet was proposed to mediate genome dimerization [47]. It was also demonstrated for Ty1 that dimerization of short RNA transcripts induced by the chaperone synthetic peptide (TYA1-D) was inefficient when the PBS was mutated and hybridization of tRNAiMet was compromised [89]. However, further studies [45] demonstrated that gRNA dimerization was not decreased in Ty3 IN mutants that failed to package tRNAiMet. Moreover, in a recent study [108] the authors monitored Ty3 packaging using a benzonase assay [74,120] and showed that deletion of 5′-3′-bipartite PBS did not prevent gRNA packaging. Therefore, either tRNAiMet is not a prerequisite for packaging or unlike in retroviruses, dimerization is not essential. Arguing against the notion that dimerization and packaging of retroviruses and retrotransposons differ significantly in this regard, Feng et al. demonstrated that the Ty1 dimers resemble those of retroviruses in that they undergo stabilization during proteolytic maturation of the VLP [44,121]. By analogy to retroviruses, and in agreement with the SHAPE reactivity profiles obtained from the Ty1 gRNA in different biological states, palindromic (PAL) sequences were suggested to mediate formation of Ty1 dimers [61,119]. Altered SHAPE reactivity patterns observed in native VLPs supported involvement of PAL residues in intermolecular interactions. Moreover, PAL sequences provide protein binding sites [61,62] (Figure 3). Ty1 Gag derivatives containing the chaperone domain (TYA1-D and CTR), in addition to Ty3 NC, promote dimerization of their respective RNAs in vitro [47,62,89].

## 8. Gag Assembly into VLPs

Ty VLPs are functionally analogous to the non-enveloped core particles of retroviruses. VLPs are stable, specific nucleoprotein structures that protect gRNA from cellular nucleases and allow its reverse transcription. Immature VLPs comprise Gag and Gag-Pol precursor proteins and two copies of gRNA, tRNAiMet, in addition to cellular RNAs and proteins (reviewed in [11,12]). Clusters of VLPs at different stages of maturation are observed in retrosomes of cells expressing a Ty1 or Ty3 element [8,23,40]. VLP maturation starts from autocatalytic cleavage of PR, which directs further processing of the Pol domain, resulting in release of RT and IN. Ty3 Gag3 is processed to mature CA, SP, and NC proteins [122] (Figure 1). Ty1 Gag processing is relatively simple and PR cleavage of Gag-p49 results in mature Gag-p45 and a 40-aa acidic peptide (p4). This peptide has not been detected in VLP preparations or cell extracts [84,85,86,87]. Both immature and mature Ty1 Gag proteins can assemble into VLPs, but display different properties and only the mature Gag form *trans*-activates transposition [88]. Although Ty VLPs are formed independently of Gag and Gag-Pol processing [123,124], this process is required for VLP maturation, reverse transcription, and integration [19,84,85,122]. The external structures of Ty1 and Ty3 VLPs are similar and do not undergo major rearrangement during maturation. However Ty1 assembly seems to be more flexible and results in accumulation of VLPs of variable sizes, ranging from 30 to 80 nm in diameter [85,125,126]. Ty3 VLP populations are more homogeneous, with diameters ranging from 25 to 52 nm, as was shown using atomic force microscopy [123]. Yeast factors are essential for formation of active VLPs but Gag can assemble into spherical particles even when expressed in a heterologous host [73,114,127].

Based on mutational analyses, Gag residues important for Ty1 VLP assembly and structure have been mapped within aa 62–114 and 340–363 [128]. In another study, the functional importance of three regions, namely aa 36–50, 239–287, and 330–350 was proposed, as they were predicted to assume an α-helical configuration [129]. Recent predictions of the Ty1 Gag structure suggest its N-terminal residues 1–172 and C-terminal residues 355–401 are disordered, while the region encompassing residues 173–354 forms α-helices [62]. The C-terminal 355–401 region was demonstrated to be necessary for interactions with Ty1 RNA [62], consistent with indirect studies in yeast [88] and results obtained with Ty1 Gag-derived peptide TYA1-D [89]. Immunological analysis suggest that the N-terminal region of Ty1 Gag forms the outer shell of VLPs, while the C-terminus, containing the RNA binding domain, is internal [130].

Similar to retroviruses [131], the CA domain of Ty3 Gag3 is a major determinant of VLP assembly [123]. Residues 86–100 of Ty3 CA are consistent with the major homology motif (MHR) characteristic of retroviral CA proteins and altering MHR composition induced similar defects in retrotransposon and retroviral particle formation [132]. Structure predictions suggest that Ty3 CA comprises smaller C-terminal domain (148–207) and a large α-helical N-terminal domain (NTD) (1–39), which contributes to the outer VLP shell [74]. The N-terminal CA domain is critical for Ty3 Gag3 multimerization and mutations of the first 100 residues inhibited VLP assembly [74,133]. The N-terminal domain of CA is able to interact with both the NTD of other CA proteins and with its C-terminal domain (CTD).

The NC domain of uncleaved Ty3 Gag3, but not mature NC, is required for gRNA packaging into VLPs since mutations disrupting mature NC production did not inhibit gRNA encapsidation [75]. The conserved zinc-finger motif is, however, critical [76]. Nevertheless, disrupting the zinc-finger motif or deleting the NC domain did not abolish Gag3 and Gag3-Pol3 multimerization and their aggregates were observed in the nucleus of cells expressing Ty3 [72]. This indicates that the NC domain of Ty3 Gag3 is critical for both gRNA trafficking and packaging into VLPs, while the CA domain may be more essential for structural stability of particles than observed for retroviruses [72,134].

The spacer domain (SP), located between CA and NC domains of Ty3 Gag3, is not critical for VLP assembly. However, deleting the SP domain results in formation of more compact VLPs that are defective for retrotransposition. The Ty3 SP domain is more acidic than known retroviral SP and substitutions of acidic residues to alanine led to VLP disruption and inhibition of retrotransposition [75]. Based on those observations, a model of SP contributing as a molecular “spring” to Ty3 VLP assembly has been proposed. During Ty3 retrosome formation, intramolecular interactions between the acidic SP domain and basic region of NC limit premature Gag3 multimerization. While the NC domain binds to gRNA, intermolecular interactions of NC and SP can occur to facilitate Gag3 multimerization on gRNA and CA-CA interactions. After Ty3 Gag3 processing, mature NC remains bound to gRNA while negatively charged SP in the CA-SP intermediate, which constitutes a significant fraction of processing products, could act as molecular “spring” and destabilize the CA-SP interactions, thereby fostering disassembly and release of cDNA.

## 9. Gag/RNA Interactions Important for Copy Number Control (CNC)

There are many mechanisms that may limit the effects of retrotransposition on the host genome. With regards to Ty RNA packaging, one such mechanism is of particular interest. Garfinkel et al. discovered Ty1 copy number control (CNC) [135] which is characterized by decreased retrotransposition when additional elements are present in the genome [136]. Structural analyses via SHAPE suggested that altered Ty1 gRNA/Gag interactions may participate in conferring CNC [61]. Recently a protein factor (p22), necessary and sufficient for CNC, has been identified [30] and its mechanism of action has been proposed. p22 is encoded by an internally initiated Ty1 mRNA and is an N-terminal truncated form of Gag that is cleaved by Ty1 PR at the same site as Gag to form p18. The multifaceted approach illuminated how this restriction factor interacts with Gag to inhibit retrotransposition [62]. Although Ty1 Gag and p22 play opposing roles in retrotransposition, they share a nucleic acid chaperone domain. p18 exhibits lower chaperone activity than Gag in tRNAiMet annealing and dimerization in vitro but binds Ty1 RNA with similarly high affinity. p18 and Gag bind within the same sites on Ty1 RNA, arguing they might compete for gRNA binding. Moreover, nuclease protection assays revealed that p22/p18 prevents stable packaging of Ty1 RNA [62]. By analyzing missense mutations in Ty1 that confer partial resistance to p22, a further study found that p22/p18 disturbs the central function of Gag during VLP assembly [137]. Therefore, Ty1 RNA packaging constitutes an important step that helps keep retrotransposition in check [136].

## 10. Conclusions

gRNA plays two distinct roles in the retrotransposon life cycle, namely as a template for translation and reverse transcription (Figure 2). gRNAs are not divided into separate pools of translated RNA and packaged RNA, therefore their trafficking and packaging into VLPs requires an equilibrium between competing events of translation and packaging. Perhaps Gag binding to RNA excludes newly assembling ribosomes and, as a consequence, leads to packaging and reverse transcription. *Cis*-acting sequences and structures important for packaging are not clearly defined and it is likely that multiple RNA sites scattered outside major packaging regions may contribute to the process. Moreover, it is not known if the same nucleotide sequences are recognized by Gag during gRNA nuclear export, trafficking to retrosomes and packaging. Many questions remain open but it is clear that RNA packaging and VLP formation is critical for Ty propagation. The VLP protects retrotransposon gRNA and concentrates all factors required for reverse transcription. VLP formation may also protect the host from high levels of free reverse transcriptase that could be potentially harmful.

## References

[1] Havecker E.R., Gao X., Voytas D.F. (2004). The diversity of LTR retrotransposons. Genome Biol..

[2] Jern P., Coffin J.M. (2008). Effects of retroviruses on host genome function. Annu. Rev. Genet..

[3] Llorens C., Fares M.A., Moya A. (2008). Relationships of gag-pol diversity between Ty3/Gypsy and retroviridae LTR retroelements and the three kings hypothesis. BMC Evol. Biol..

[4] Malik H.S., Henikoff S., Eickbush T.H. (2000). Poised for contagion: Evolutionary origins of the infectious abilities of invertebrate retroviruses. Genome Res..

[5] Voytas D.F., Boeke J.D., Craig N.L., Craigie R., Gellert M., Lambowitz A.M. (2002). Ty1 and Ty5 of *Sacharomyces cerevisiae*. Mobile DNA II.

[6] Boeke J.D., Garfinkel D.J., Styles C.A., Fink G.R. (1985). Ty elements transpose through an RNA intermediate. Cell.

[7] Clark D.J., Bilanchone V.W., Haywood L.J., Dildine S.L., Sandmeyer S.B. (1988). A yeast sigma composite element, Ty3, has properties of a retrotransposon. J. Biol. Chem..

[8] Garfinkel D.J., Boeke J.D., Fink G.R. (1985). Ty element transposition: Reverse transcriptase and virus-like particles. Cell.

[9] Garfinkel D.J. (2005). Genome evolution mediated by ty elements in *Saccharomyces*. Cytogenet. Genome Res..

[10] Mita P., Boeke J.D. (2016). How retrotransposons shape genome regulation. Curr. Opin. Genet. Dev..

[11] Curcio M.J., Lutz S., Lesage P. (2015). The Ty1 LTR-retrotransposon of budding yeast. Microbiol. Spectr..

[12] Sandmeyer S., Patterson K., Bilanchone V. (2015). Ty3, a position-specific retrotransposon in budding yeast. Microbiol. Spectr..

[13] Curcio M.J., Garfinkel D.J. (1994). Heterogeneous functional Ty1 elements are abundant in the saccharomyces cerevisiae genome. Genetics.

[14] Elder R.T., St John T.P., Stinchcomb D.T., Davis R.W., Scherer S., Davis R.W. (1981). Studies on the transposable element Ty1 of yeast. I. RNA homologous to Ty1. II. Recombination and expression of Ty1 and adjacent sequences. Cold Spring Harb. Symp. Quant. Biol..

[15] Carr M., Bensasson D., Bergman C.M. (2012). Evolutionary genomics of transposable elements in saccharomyces cerevisiae. PLoS ONE.

[16] Bilanchone V.W., Claypool J.A., Kinsey P.T., Sandmeyer S.B. (1993). Positive and negative regulatory elements control expression of the yeast retrotransposon Ty3. Genetics.

[17] Hansen L.J., Chalker D.L., Sandmeyer S.B. (1988). Ty3, a yeast retrotransposon associated with tRNA genes, has homology to animal retroviruses. Mol. Cell. Biol..

[18] Menees T.M., Sandmeyer S.B. (1994). Transposition of the yeast retroviruslike element Ty3 is dependent on the cell cycle. Mol. Cell. Biol..

[19] Mellor J., Fulton A.M., Dobson M.J., Roberts N.A., Wilson W., Kingsman A.J., Kingsman S.M. (1985). The Ty transposon of saccharomyces cerevisiae determines the synthesis of at least three proteins. Nucleic Acids Res..

[20] Elder R.T., Loh E.Y., Davis R.W. (1983). RNA from the yeast transposable element Ty1 has both ends in the direct repeats, a structure similar to retrovirus RNA. Proc. Natl. Acad. Sci. USA.

[21] Mules E.H., Uzun O., Gabriel A. (1998). In vivo Ty1 reverse transcription can generate replication intermediates with untidy ends. J. Virol..

[22] Eickbush T.H., Jamburuthugoda V.K. (2008). The diversity of retrotransposons and the properties of their reverse transcriptases. Virus Res..

[23] Hansen L.J., Chalker D.L., Orlinsky K.J., Sandmeyer S.B. (1992). Ty3 Gag3 and POL3 genes encode the components of intracellular particles. J. Virol..

[24] Hansen L.J., Sandmeyer S.B. (1990). Characterization of a transpositionally active Ty3 element and identification of the Ty3 integrase protein. J. Virol..

[25] Curcio M.J., Hedge A.M., Boeke J.D., Garfinkel D.J. (1990). Ty RNA levels determine the spectrum of retrotransposition events that activate gene expression in saccharomyces cerevisiae. Mol. Gen. Genet..

[26] Curcio M.J., Sanders N.J., Garfinkel D.J. (1988). Transpositional competence and transcription of endogenous ty elements in *Saccharomyces cerevisiae*: Implications for regulation of transposition. Mol. Cell. Biol..

[27] Munchel S.E., Shultzaberger R.K., Takizawa N., Weis K. (2011). Dynamic profiling of mRNA turnover reveals gene-specific and system-wide regulation of mRNA decay. Mol. Biol. Cell.

[28] Checkley M.A., Mitchell J.A., Eizenstat L.D., Lockett S.J., Garfinkel D.J. (2013). Ty1 gag enhances the stability and nuclear export of Ty1 mRNA. Traffic.

[29] Berretta J., Pinskaya M., Morillon A. (2008). A cryptic unstable transcript mediates transcriptional trans-silencing of the Ty1 retrotransposon in *S. cerevisiae*. Genes Dev..

[30] Saha A., Mitchell J.A., Nishida Y., Hildreth J.E., Ariberre J.A., Gilbert W.V., Garfinkel D.J. (2015). A trans-dominant form of gag restricts Ty1 retrotransposition and mediates copy number control. J. Virol..

[31] Winston F., Durbin K.J., Fink G.R. (1984). The *SPT3* gene is required for normal transcription of Ty elements in *S. cerevisiae*. Cell.

[32] Yu K., Elder R.T. (1989). Some of the signals for 3′-end formation in transcription of the *Saccharomyces cerevisiae* Ty-d15 element are immediately downstream of the initiation site. Mol. Cell. Biol..

[33] Belcourt M.F., Farabaugh P.J. (1990). Ribosomal frameshifting in the yeast retrotransposon Ty: TRNAs induce slippage on a 7 nucleotide minimal site. Cell.

[34] Clare J.J., Belcourt M., Farabaugh P.J. (1988). Efficient translational frameshifting occurs within a conserved sequence of the overlap between the two genes of a yeast Ty1 transposon. Proc. Natl. Acad. Sci. USA.

[35] Farabaugh P.J., Zhao H., Vimaladithan A. (1993). A novel programed frameshift expresses the POL3 gene of retrotransposon Ty3 of yeast: Frameshifting without tRNA slippage. Cell.

[36] Kirchner J., Sandmeyer S.B., Forrest D.B. (1992). Transposition of a Ty3 GAG3-POL3 fusion mutant is limited by availability of capsid protein. J. Virol..

[37] Vimaladithan A., Farabaugh P.J. (1994). Special peptidyl-tRNA molecules can promote translational frameshifting without slippage. Mol. Cell. Biol..

[38] Kawakami K., Pande S., Faiola B., Moore D.P., Boeke J.D., Farabaugh P.J., Strathern J.N., Nakamura Y., Garfinkel D.J. (1993). A rare tRNA-Arg(CCU) that regulates Ty1 element ribosomal frameshifting is essential for Ty1 retrotransposition in *Saccharomyces cerevisiae*. Genetics.

[39] Xu H., Boeke J.D. (1990). Host genes that influence transposition in yeast: The abundance of a rare tRNA regulates Ty1 transposition frequency. Proc. Natl. Acad. Sci. USA.

[40] Beliakova-Bethell N., Beckham C., Giddings T.H., Winey M., Parker R., Sandmeyer S. (2006). Virus-like particles of the Ty3 retrotransposon assemble in association with P-body components. RNA.

[41] Malagon F., Jensen T.H. (2008). The T body, a new cytoplasmic RNA granule in *Saccharomyces cerevisiae*. Mol. Cell. Biol..

[42] Malagon F., Jensen T.H. (2011). T-body formation precedes virus-like particle maturation in *S. cerevisiae*. RNA Biol..

[43] Sandmeyer S.B., Clemens K.A. (2010). Function of a retrotransposon nucleocapsid protein. RNA Biol..

[44] Feng Y.X., Moore S.P., Garfinkel D.J., Rein A. (2000). The genomic RNA in Ty1 virus-like particles is dimeric. J. Virol..

[45] Nymark-McMahon M.H., Beliakova-Bethell N.S., Darlix J.L., le Grice S.F., Sandmeyer S.B. (2002). Ty3 integrase is required for initiation of reverse transcription. J. Virol..

[46] Chapman K.B., Bystrom A.S., Boeke J.D. (1992). Initiator methionine tRNA is essential for Ty1 transposition in yeast. Proc. Natl. Acad. Sci. USA.

[47] Gabus C., Ficheux D., Rau M., Keith G., Sandmeyer S., Darlix J.L. (1998). The yeast Ty3 retrotransposon contains a 5′-3′ bipartite primer-binding site and encodes nucleocapsid protein NCp9 functionally homologous to HIV-1 NCp7. EMBO J..

[48] Keeney J.B., Chapman K.B., Lauermann V., Voytas D.F., Aström S.U., von Pawel-Rammingen U., Bystrom A., Boeke J.D. (1995). Multiple molecular determinants for retrotransposition in a primer tRNA. Mol. Cell. Biol..

[49] Kenna M.A., Brachmann C.B., Devine S.E., Boeke J.D. (1998). Invading the yeast nucleus: A nuclear localization signal at the C terminus of Ty1 integrase is required for transposition in vivo. Mol. Cell. Biol..

[50] Lin S.S., Nymark-McMahon M.H., Yieh L., Sandmeyer S.B. (2001). Integrase mediates nuclear localization of Ty3. Mol. Cell. Biol..

[51] Devine S.E., Boeke J.D. (1996). Integration of the yeast retrotransposon Ty1 is targeted to regions upstream of genes transcribed by RNA polymerase III. Genes Dev..

[52] Eigel A., Feldmann H. (1982). Ty1 and delta elements occur adjacent to several tRNA genes in yeast. EMBO J..

[53] Kim J.M., Vanguri S., Boeke J.D., Gabriel A., Voytas D.F. (1998). Transposable elements and genome organization: A comprehensive survey of retrotransposons revealed by the complete saccharomyces cerevisiae genome sequence. Genome Res..

[54] Qi X., Daily K., Nguyen K., Wang H., Mayhew D., Rigor P., Forouzan S., Johnston M., Mitra R.D., Baldi P. (2012). Retrotransposon profiling of RNA polymerase III initiation sites. Genome Res..

[55] Connolly C.M., Sandmeyer S.B. (1997). RNA polymerase III interferes with Ty3 integration. FEBS Lett..

[56] Kirchner J., Connolly C.M., Sandmeyer S.B. (1995). Requirement of RNA polymerase iii transcription factors for in vitro position-specific integration of a retroviruslike element. Science.

[57] Yieh L., Hatzis H., Kassavetis G., Sandmeyer S.B. (2002). Mutational analysis of the transcription factor IIIB-DNA target of Ty3 retroelement integration. J. Biol. Chem..

[58] Yieh L., Kassavetis G., Geiduschek E.P., Sandmeyer S.B. (2000). The Brf and TATA-binding protein subunits of the RNA polymerase III transcription factor IIIB mediate position-specific integration of the gypsy-like element, Ty3. J. Biol. Chem..

[59] Bridier-Nahmias A., Tchalikian-Cosson A., Baller J.A., Menouni R., Fayol H., Flores A., Saib A., Werner M., Voytas D.F., Lesage P. (2015). Retrotransposons. An RNA polymerase III subunit determines sites of retrotransposon integration. Science.

[60] Xu H., Boeke J.D. (1990). Localization of sequences required in *cis* for yeast Ty1 element transposition near the long terminal repeats: Analysis of mini-Ty1 elements. Mol. Cell. Biol..

[61] Purzycka K.J., Legiewicz M., Matsuda E., Eizentstat L.D., Lusvarghi S., Saha A., le Grice S.F., Garfinkel D.J. (2013). Exploring Ty1 retrotransposon RNA structure within virus-like particles. Nucleic Acids Res..

[62] Nishida Y., Pachulska-Wieczorek K., Blaszczyk L., Saha A., Gumna J., Garfinkel D.J., Purzycka K.J. (2015). Ty1 retrovirus-like element Gag contains overlapping restriction factor and nucleic acid chaperone functions. Nucleic Acids Res..

[63] Cristofari G., Bampi C., Wilhelm M., Wilhelm F.X., Darlix J.L. (2002). A 5′-3′ long-range interaction in Ty1 RNA controls its reverse transcription and retrotransposition. EMBO J..

[64] Friant S., Heyman T., Wilhelm M.L., Wilhelm F.X. (1996). Extended interactions between the primer tRNAi(Met) and genomic RNA of the yeast Ty1 retrotransposon. Nucleic Acids Res..

[65] Wilhelm M., Wilhelm F.X., Keith G., Agoutin B., Heyman T. (1994). Yeast Ty1 retrotransposon: The minus-strand primer binding site and a *cis*-acting domain of the Ty1 RNA are both important for packaging of primer tRNA inside virus-like particles. Nucleic Acids Res..

[66] Friant S., Heyman T., Bystrom A.S., Wilhelm M., Wilhelm F.X. (1998). Interactions between Ty1 retrotransposon RNA and the T and D regions of the tRNA(iMet) primer are required for initiation of reverse transcription in vivo. Mol. Cell. Biol..

[67] Bolton E.C., Coombes C., Eby Y., Cardell M., Boeke J.D. (2005). Identification and characterization of critical *cis*-acting sequences within the yeast Ty1 retrotransposon. RNA.

[68] Huang Q., Purzycka K.J., Lusvarghi S., Li D., Legrice S.F., Boeke J.D. (2013). Retrotransposon Ty1 RNA contains a 5′-terminal long-range pseudoknot required for efficient reverse transcription. RNA.

[69] Purzycka K.J., Pachulska-Wieczorek K., Adamiak R.W. (2011). The in vitro loose dimer structure and rearrangements of the HIV-2 leader RNA. Nucleic Acids Res..

[70] Watts J.M., Dang K.K., Gorelick R.J., Leonard C.W., Bess J.W., Swanstrom R., Burch C.L., Weeks K.M. (2009). Architecture and secondary structure of an entire HIV-1 RNA genome. Nature.

[71] Kertesz M., Wan Y., Mazor E., Rinn J.L., Nutter R.C., Chang H.Y., Segal E. (2010). Genome-wide measurement of RNA secondary structure in yeast. Nature.

[72] Larsen L.S., Beliakova-Bethell N., Bilanchone V., Zhang M., Lamsa A., Dasilva R., Hatfield G.W., Nagashima K., Sandmeyer S. (2008). Ty3 nucleocapsid controls localization of particle assembly. J. Virol..

[73] Larsen L.S., Kuznetsov Y., McPherson A., Hatfield G.W., Sandmeyer S. (2008). TY3 *GAG3* protein forms ordered particles in *Escherichia coli*. Virology.

[74] Larsen L.S., Zhang M., Beliakova-Bethell N., Bilanchone V., Lamsa A., Nagashima K., Najdi R., Kosaka K., Kovacevic V., Cheng J. (2007). Ty3 capsid mutations reveal early and late functions of the amino-terminal domain. J. Virol..

[75] Clemens K., Larsen L., Zhang M., Kuznetsov Y., Bilanchone V., Randall A., Harned A., Dasilva R., Nagashima K., McPherson A. (2011). The Ty3 Gag3 spacer controls intracellular condensation and uncoating. J. Virol..

[76] Orlinsky K.J., Sandmeyer S.B. (1994). The Cys-His motif of Ty3 NC can be contributed by Gag3 or Gag3-Pol3 polyproteins. J. Virol..

[77] Mirambeau G., Lyonnais S., Gorelick R.J. (2010). Features, processing states, and heterologous protein interactions in the modulation of the retroviral nucleocapsid protein function. RNA Biol..

[78] Chaurasiya K.R., Geertsema H., Cristofari G., Darlix J.L., Williams M.C. (2012). A single zinc finger optimizes the DNA interactions of the nucleocapsid protein of the yeast retrotransposon Ty3. Nucleic Acids Res..

[79] Rein A. (2010). Nucleic acid chaperone activity of retroviral Gag proteins. RNA Biol..

[80] Feng Y.X., Campbell S., Harvin D., Ehresmann B., Ehresmann C., Rein A. (1999). The human immunodeficiency virus Type 1 Gag polyprotein has nucleic acid chaperone activity: Possible role in dimerization of genomic RNA and placement of tRNA on the primer binding site. J. Virol..

[81] Pachulska-Wieczorek K., Blaszczyk L., Biesiada M., Adamiak R.W., Purzycka K.J. (2016). The matrix domain contributes to the nucleic acid chaperone activity of HIV-2 Gag. Retrovirology.

[82] Wu T., Datta S.A., Mitra M., Gorelick R.J., Rein A., Levin J.G. (2010). Fundamental differences between the nucleic acid chaperone activities of HIV-1 nucleocapsid protein and Gag or Gag-derived proteins: Biological implications. Virology.

[83] Adams S.E., Mellor J., Gull K., Sim R.B., Tuite M.F., Kingsman S.M., Kingsman A.J. (1987). The functions and relationships of Ty-VLP proteins in yeast reflect those of mammalian retroviral proteins. Cell.

[84] Garfinkel D.J., Hedge A.M., Youngren S.D., Copeland T.D. (1991). Proteolytic processing of pol-TYB proteins from the yeast retrotransposon Ty1. J. Virol..

[85] HA A.L.-K., Bhella D., Kenney J.M., Roth J.F., Kingsman A.J., Martin-Rendon E., Saibil H.R. (1999). Yeast Ty retrotransposons assemble into virus-like particles whose T-numbers depend on the C-terminal length of the capsid protein. J. Mol. Biol..

[86] Merkulov G.V., Swiderek K.M., Brachmann C.B., Boeke J.D. (1996). A critical proteolytic cleavage site near the C terminus of the yeast retrotransposon Ty1 Gag protein. J. Virol..

[87] Youngren S.D., Boeke J.D., Sanders N.J., Garfinkel D.J. (1988). Functional organization of the retrotransposon Ty from saccharomyces cerevisiae: Ty protease is required for transposition. Mol. Cell. Biol..

[88] Roth J.F., Kingsman S.M., Kingsman A.J., Martin-Rendon E. (2000). Possible regulatory function of the *Saccharomyces cerevisiae* Ty1 retrotransposon core protein. Yeast.

[89] Cristofari G., Ficheux D., Darlix J.L. (2000). The Gag-like protein of the yeast Ty1 retrotransposon contains a nucleic acid chaperone domain analogous to retroviral nucleocapsid proteins. J. Biol. Chem..

[90] De Rocquigny H., Gabus C., Vincent A., Fournie-Zaluski M.C., Roques B., Darlix J.L. (1992). Viral RNA annealing activities of human immunodeficiency virus type 1 nucleocapsid protein require only peptide domains outside the zinc fingers. Proc. Natl. Acad. Sci. USA.

[91] Housset V., De Rocquigny H., Roques B.P., Darlix J.L. (1993). Basic amino acids flanking the zinc finger of moloney murine leukemia virus nucleocapsid protein NCp10 are critical for virus infectivity. J. Virol..

[92] Le Cam E., Coulaud D., Delain E., Petitjean P., Roques B.P., Gerard D., Stoylova E., Vuilleumier C., Stoylov S.P., Mely Y. (1998). Properties and growth mechanism of the ordered aggregation of a model RNA by the HIV-1 nucleocapsid protein: An electron microscopy investigation. Biopolymers.

[93] Pachulska-Wieczorek K., Stefaniak A.K., Purzycka K.J. (2014). Similarities and differences in the nucleic acid chaperone activity of HIV-2 and HIV-1 nucleocapsid proteins in vitro. Retrovirology.

[94] Wu H., Mitra M., Naufer M.N., McCauley M.J., Gorelick R.J., Rouzina I., Musier-Forsyth K., Williams M.C. (2014). Differential contribution of basic residues to HIV-1 nucleocapsid protein’s nucleic acid chaperone function and retroviral replication. Nucleic Acids Res..

[95] Doh J.H., Lutz S., Curcio M.J. (2014). Co-translational localization of an LTR-retrotransposon RNA to the endoplasmic reticulum nucleates virus-like particle assembly sites. PLoS Genet..

[96] Hermesh O., Jansen R.P. (2013). Take the (RN)a-train: Localization of mRNA to the endoplasmic reticulum. Biochim. Biophys. Acta.

[97] Checkley M.A., Nagashima K., Lockett S.J., Nyswaner K.M., Garfinkel D.J. (2010). P-body components are required for Ty1 retrotransposition during assembly of retrotransposition-competent virus-like particles. Mol. Cell. Biol..

[98] Dupont S., Sharova N., DeHoratius C., Virbasius C.M., Zhu X., Bukrinskaya A.G., Stevenson M., Green M.R. (1999). A novel nuclear export activity in HIV-1 matrix protein required for viral replication. Nature.

[99] Nash M.A., Meyer M.K., Decker G.L., Arlinghaus R.B. (1993). A subset of Pr65gag is nucleus associated in murine leukemia virus-infected cells. J. Virol..

[100] Schliephake A.W., Rethwilm A. (1994). Nuclear localization of foamy virus Gag precursor protein. J. Virol..

[101] Scheifele L.Z., Garbitt R.A., Rhoads J.D., Parent L.J. (2002). Nuclear entry and CRM1-dependent nuclear export of the Rous sarcoma virus Gag polyprotein. Proc. Natl. Acad. Sci. USA.

[102] Garbitt-Hirst R., Kenney S.P., Parent L.J. (2009). Genetic evidence for a connection between Rous sarcoma virus Gag nuclear trafficking and genomic RNA packaging. J. Virol..

[103] Aizer A., Brody Y., Ler L.W., Sonenberg N., Singer R.H., Shav-Tal Y. (2008). The dynamics of mammalian P body transport, assembly, and disassembly in vivo. Mol. Biol. Cell.

[104] Anderson P., Kedersha N. (2009). RNA granules: Post-transcriptional and epigenetic modulators of gene expression. Nat. Rev. Mol. Cell. Biol..

[105] Buchan J.R., Muhlrad D., Parker R. (2008). P bodies promote stress granule assembly in saccharomyces cerevisiae. J. Cell Biol..

[106] Brengues M., Teixeira D., Parker R. (2005). Movement of eukaryotic mRNAs between polysomes and cytoplasmic processing bodies. Science.

[107] Teixeira D., Sheth U., Valencia-Sanchez M.A., Brengues M., Parker R. (2005). Processing bodies require RNA for assembly and contain nontranslating mRNAs. RNA.

[108] Clemens K., Bilanchone V., Beliakova-Bethell N., Larsen L.S., Nguyen K., Sandmeyer S. (2013). Sequence requirements for localization and packaging of Ty3 retroelement RNA. Virus Res..

[109] Bilanchone V., Clemens K., Kaake R., Dawson A.R., Matheos D., Nagashima K., Sitlani P., Patterson K., Chang I., Huang L. (2015). Ty3 retrotransposon hijacks mating yeast RNA processing bodies to infect new genomes. PLoS Genet..

[110] Dutko J.A., Kenny A.E., Gamache E.R., Curcio M.J. (2010). 5′ to 3′ mRNA decay factors colocalize with Ty1 gag and human APOBEC3G and promote Ty1 retrotransposition. J. Virol..

[111] Irwin B., Aye M., Baldi P., Beliakova-Bethell N., Cheng H., Dou Y., Liou W., Sandmeyer S. (2005). Retroviruses and yeast retrotransposons use overlapping sets of host genes. Genome Res..

[112] Griffith J.L., Coleman L.E., Raymond A.S., Goodson S.G., Pittard W.S., Tsui C., Devine S.E. (2003). Functional genomics reveals relationships between the retrovirus-like Ty1 element and its host *Saccharomyces cerevisiae*. Genetics.

[113] Mellor J., Malim M.H., Gull K., Tuite M.F., McCready S., Dibbayawan T., Kingsman S.M., Kingsman A.J. (1985). Reverse transcriptase activity and Ty RNA are associated with virus-like particles in yeast. Nature.

[114] Luschnig C., Bachmair A. (1997). RNA packaging of yeast retrotransposon Ty1 in the heterologous host, *Escherichia coli*. Biol. Chem..

[115] Lu K., Heng X., Summers M.F. (2011). Structural determinants and mechanism of HIV-1 genome packaging. J. Mol. Biol..

[116] D’Souza V., Summers M.F. (2005). How retroviruses select their genomes. Nat. Rev. Microbiol..

[117] Kutluay S.B., Zang T., Blanco-Melo D., Powell C., Jannain D., Errando M., Bieniasz P.D. (2014). Global changes in the RNA binding specificity of HIV-1 gag regulate virion genesis. Cell.

[118] Rein A., Datta S.A., Jones C.P., Musier-Forsyth K. (2011). Diverse interactions of retroviral gag proteins with RNAs. Trends Biochem. Sci..

[119] Purzycka K.J., Garfinkel D.J., Boeke J.D., le Grice S.F. (2013). Influence of RNA structural elements on Ty1 retrotransposition. Mob. Genet. Elem..

[120] Lin J.H., Levin H.L. (1998). Reverse transcription of a self-primed retrotransposon requires an RNA structure similar to the U5-IR stem-loop of retroviruses. Mol. Cell. Biol..

[121] Shehu-Xhilaga M., Kraeusslich H.G., Pettit S., Swanstrom R., Lee J.Y., Marshall J.A., Crowe S.M., Mak J. (2001). Proteolytic processing of the p2/nucleocapsid cleavage site is critical for human immunodeficiency virus type 1 RNA dimer maturation. J. Virol..

[122] Kirchner J., Sandmeyer S. (1993). Proteolytic processing of Ty3 proteins is required for transposition. J. Virol..

[123] Kuznetsov Y.G., Zhang M., Menees T.M., McPherson A., Sandmeyer S. (2005). Investigation by atomic force microscopy of the structure of Ty3 retrotransposon particles. J. Virol..

[124] Muller F., Bruhl K.H., Freidel K., Kowallik K.V., Ciriacy M. (1987). Processing of Ty1 proteins and formation of Ty1 virus-like particles in saccharomyces cerevisiae. Mol. Gen. Genet..

[125] Burns N.R., Saibil H.R., White N.S., Pardon J.F., Timmins P.A., Richardson S.M., Richards B.M., Adams S.E., Kingsman S.M., Kingsman A.J. (1992). Symmetry, flexibility and permeability in the structure of yeast retrotransposon virus-like particles. EMBO J..

[126] Palmer K.J., Tichelaar W., Myers N., Burns N.R., Butcher S.J., Kingsman A.J., Fuller S.D., Saibil H.R. (1997). Cryo-electron microscopy structure of yeast Ty retrotransposon virus-like particles. J. Virol..

[127] Luschnig C., Hess M., Pusch O., Brookman J., Bachmair A. (1995). The gag homologue of retrotransposon Ty1 assembles into spherical particles in *Escherichia coli*. Eur. J. Biochem..

[128] Brookman J.L., Stott A.J., Cheeseman P.J., Adamson C.S., Holmes D., Cole J., Burns N.R. (1995). Analysis of TYA protein regions necessary for formation of the Ty1 virus-like particle structure. Virology.

[129] Martin-Rendon E., Marfany G., Wilson S., Ferguson D.J.P., Kingsman S.M., Kingsman A.J. (1996). Structural determinants within the subunit protein of Ty1 virus-like particles. Mol. Microbiol..

[130] Brookman J.L., Stott A.J., Cheeseman P.J., Burns N.R., Adams S.E., Kingsman A.J., Gull K. (1995). An immunological analysis of Ty1 virus-like particle structure. Virology.

[131] Ganser-Pornillos B.K., Yeager M., Pornillos O. (2012). Assembly and architecture of HIV. Adv. Exp. Med. Biol..

[132] Orlinsky K.J., Gu J., Hoyt M., Sandmeyer S., Menees T.M. (1996). Mutations in the Ty3 major homology region affect multiple steps in Ty3 retrotransposition. J. Virol..

[133] Zhang M., Larsen L.S., Irwin B., Bilanchone V., Sandmeyer S. (2010). Two-hybrid analysis of Ty3 capsid subdomain interactions. Mob. DNA.

[134] Wang S.W., Noonan K., Aldovini A. (2004). Nucleocapsid-RNA interactions are essential to structural stability but not to assembly of retroviruses. J. Virol..

[135] Garfinkel D.J., Nyswaner K., Wang J., Cho J.Y. (2003). Post-transcriptional cosuppression of Ty1 retrotransposition. Genetics.

[136] Garfinkel D.J., Tucker J.M., Saha A., Nishida Y., Pachulska-Wieczorek K., Błaszczyk L., Purzycka K.J. (2016). A self-encoded capsid derivative restricts Ty1 retrotransposition in *Saccharomyces*. Curr. Genet..

[137] Tucker J.M., Larango M.E., Wachsmuth L.P., Kannan N., Garfinkel D.J. (2015). The Ty1 retrotransposon restriction factor p22 targets Gag. PLoS Genet..

